# Complete Genome Sequence of a Novel Human Enterovirus 85 (HEV85) Recombinant with an Unknown New Serotype HEV-B Donor Sequence Isolated from a Child with Acute Flaccid Paralysis

**DOI:** 10.1128/genomeA.00015-12

**Published:** 2013-01-15

**Authors:** Qiang Sun, Yong Zhang, Hui Cui, Shuangli Zhu, Zhen Zhu, Guohong Huang, Xiaolei Li, Bo Zhang, Dongmei Yan, Hongqiu An, Wenbo Xu

**Affiliations:** a WHO WPRO Regional Polio Reference Laboratory, National Institute for Viral Disease Control and Prevention, Chinese Center for Disease Control and Prevention, Beijing, People’s Republic of China; b Xinjiang Uygur Autonomous Region Center for Disease Control and Prevention, Urumqi, Xinjiang Uygur Autonomous Region, People’s Republic of China

## Abstract

A Chinese human enterovirus 85 (HEV85) isolate, HTYT-ARL-AFP02F/XJ/CHN/2011, was isolated from a stool specimen of a child with acute flaccid paralysis in Xinjiang, China, in 2011. The complete genome sequence revealed that a natural intertypic recombination event had occurred between HEV85 and a previously undescribed serotype of HEV-B.

## GENOME ANNOUNCEMENT

Human enteroviruses (HEVs) are members of the genus *Enterovirus* within the family Picornaviridae, order Picornavirales, and include four species: HEV-A, HEV-B, HEV-C, and HEV-D (1). HEV85 belongs to the HEV-B species, which currently consists of 60 serotypes. HEV-B viruses are usually associated with the pathology of diseases such as acute aseptic meningitis; acute flaccid paralysis (AFP); hand, foot, and mouth disease; and viral myocarditis (2–5).

The prototype strain of HEV85, strain BAN00-10353/BAN/2000, was identified in a stool sample from a patient who had presented with AFP in Bangladesh in 2000 (6). At present, only a single nucleotide sequence of HEV85 (the complete genome sequence of the prototype strain) is available in the GenBank database. We report a complete genome sequence of a Chinese HEV85 strain (named HTYT-ARL-AFP02F/XJ/CHN/2011). This strain was isolated from a stool specimen of a patient with AFP in the Xinjiang Uygur Autonomous Region of China in 2011 during AFP surveillance activities conducted in support of global polio eradication.

The complete genome sequence of the Chinese HEV85 isolate was acquired according to the published strategies for HEV sequencing after purification by plaque assay (7–9). Raw sequence data were assembled using Sequencher software (version 4.0.5). Sequence alignments and phylogenetic trees were generated using the MEGA program (version 5.0) (10), whereas the similarity plot was generated and Bootscan analysis was performed using the SimPlot program (version 3.5.1) (11).

The genome organization of the Chinese HEV85 strain is similar to those of the other reported HEV genomes. It is 7,423 nucleotides (nt) long and is composed of a single, large open reading frame of 6,579 nt that encodes a polyprotein of 2,191 amino acids. The nucleotide and amino acid sequence similarities between the Chinese HEV85 strain and the prototype strain were 86.66% and 98.08%, respectively. Phylogenetic analysis showed that they were clustered with the HEV85 prototype strain for the *P1* coding region, but it did not show high sequence homology with the *P2* and *P3* coding regions. Furthermore, the high bootstrap value obtained in Bootscan analyses strongly suggests that recombination events occurred between HEV85 and a new serotype HEV-B that has not been previously described. The likely crossover site appears to be after nt 3370 in the 2A region. These findings highlight that recombination is a common phenomenon occurring in HEVs (12–15) and that many novel serotype HEVs remain to be isolated and described.

Together with the isolation of HEV85, a novel and recently identified HEV serotype, the trapping of an unknown new serotype HEV-B donor sequence in the Chinese HEV85 recombinant described in this study suggests that new HEV-B serotypes may be in circulation in the Xinjiang region of China. Hence, we have increased HEV surveillance in the Xinjiang region to determine the precise donor sequence of the new serotype HEV.

### Nucleotide sequence accession number. 

The nucleotide sequence of the complete genome of HEV85 recombinant strain HTYT-ARL-AFP02F/XJ/CHN/2011 has been submitted to GenBank under accession number JX898909.
